# Bilateral Lower Limb Training for Post-stroke Survivors: A Bibliometric Analysis

**DOI:** 10.7759/cureus.29615

**Published:** 2022-09-26

**Authors:** Pallavi Harjpal, Moh'd Irshad Qureshi, Rakesh K Kovela, Moli Jain

**Affiliations:** 1 Physiotherapy, Ravi Nair Physiotherapy College, Datta Meghe Institute of Medical Sciences, Wardha, IND; 2 Neuro-Physiotherapy, Ravi Nair Physiotherapy College, Datta Meghe Institute of Medical Sciences, Wardha, IND; 3 Physiotherapy, Nitte Institute of Physiotherapy, NITTE (Deemed to be University), Mangalore, IND

**Keywords:** bibliometric analysis, rehabilitation, unilateral training, bilateral training, stroke

## Abstract

Stroke is one of the most disabling conditions affecting the middle-aged population all around the world. This study aims to explore the rehabilitation of stroke patients using bibliometric analysis, which includes statistical analysis of recent articles, books, and other kinds of publications, to assess scientific output and determine the significance of scientific investigations in terms of both quality and quantity. In this study, an analysis of global trends in research in bilateral lower limb training for training balance and walking for patients in the subacute stage post-stroke between 1988 and 2021 was done. All the articles were obtained from PubMed databases. CiteSpace software was used to analyze the relationship between publications and country, journals, institutions, authors, references, and the keywords used. A total of 160 publications were included in the analysis. There was a tremendous increase in the research of physiotherapy intervention in patients who had residual disability post-stroke with a publication rate of 7.1 articles per year of publications. The use of the sophisticated PubMed database to extract articles allowed for a thorough and powerful bibliometric analysis of stroke rehabilitation research published between 1988 and 2020. In general, the number of studies on bilateral training has increased in recent decades. This historical overview of rehabilitation for post-stroke survivors will serve as a valuable starting point for future study into possible collaborators, focus issues, and trends. This bibliometric analysis highlights the potential value of exercise therapy for stroke survivors in creating more effective hemiplegia rehabilitation programs. This research may encourage the use of strengthening in the therapeutic therapy of hemiplegia balance. The groundwork will be laid for future research on strengthening stroke to be organized and given top priority.

## Introduction and background

Stroke as defined by the World Health Organization is “Fast-developing clinical evidence of localized (or general) brain dysfunction, with symptoms lasting 24 hours or more or leading to death, with no obvious etiology other than vascular origin” [1,2]. Its prevalence is increasing day by day and has reached to a point that 0.84 individuals out of 1,000 have a high risk of getting a stroke, leading to hemiplegia and hemiparesis [3-7]. Post-stroke the weakness leads to difficulty in performing daily activities and deteriorating the life of the individual. Early rehabilitation not only provides quality of life but also increases longevity [8,9].

As there is affection on the opposite side of the lesion, the unaffected side is supposed to be having no change in its performance [8,10,11]. But, in reality, it is not so, it is also affected, but less than the opposite one [12]. There is bilateral paralysis due to the uncrossed fibers affecting the overall individual’s life. Training the individual in a task-oriented pattern along with strengthening not only helps in improving balance but also makes the person ambulate independently [13-15].

For the hemi patients, while planning any protocol, we focus on the affected side, thus the other side goes into further deterioration [16-19]. Training both sides equally, although focusing on the affected side helps to improve the overall well-being of the individual [20]. There are many studies done for the upper limb for the same objective, but there is ample gap for the lower limb. Thus, this research will milestone in its own way.

## Review

Methods

Search Strategy

The data for this study came from articles published between 1988 and 2021 in PubMed, Scopus, Web of Science, Cochrane, and Cinhal databases. Because the database is still open, a comprehensive internet search was conducted on April 12, 2021, to eliminate deviation due to updating. The key terms for the search were from PubMed's Medical Subject Headings (MeSH). The keywords used for the search were: Post Stroke survivors, Bilateral training, Unilateral training, Dynamic Gait Index, and Fugl Mayer Assessment.

Analysis Tools

The data was analyzed using CiteSpace 5.3.R4, Microsoft Excel 2016, and IBM SPSS Statistics 20.0 software. The Java-based CiteSpace 5.3.R4 program is commonly used to display and analyze networks. The software's most significant feature is the creation of visual knowledge maps of authors, countries, institutions, and references. The maps are made up of various nodes and linkages. Data from the PubMed core database was tabulated in Microsoft Excel 2016 to create a trending figure of publication quantity over time. Pearson's correlation analysis of year and publication quantity was also performed using the SPSS Statistics 20.0 program (IBM Corp., Armonk, NY).

Data Extraction

Using the analysis tool, the relational figures and tables are obtained by data interpretation. The following aspects were used to interpret global research findings on exercise therapies for stroke rehabilitation: Evaluation of collaboration between nations/institutions/authors, the study of distribution and trend by journals, years, countries, institutions, authors, references, and keywords are analyzed.

Results

Analysis of the Authors

**Figure 1:**
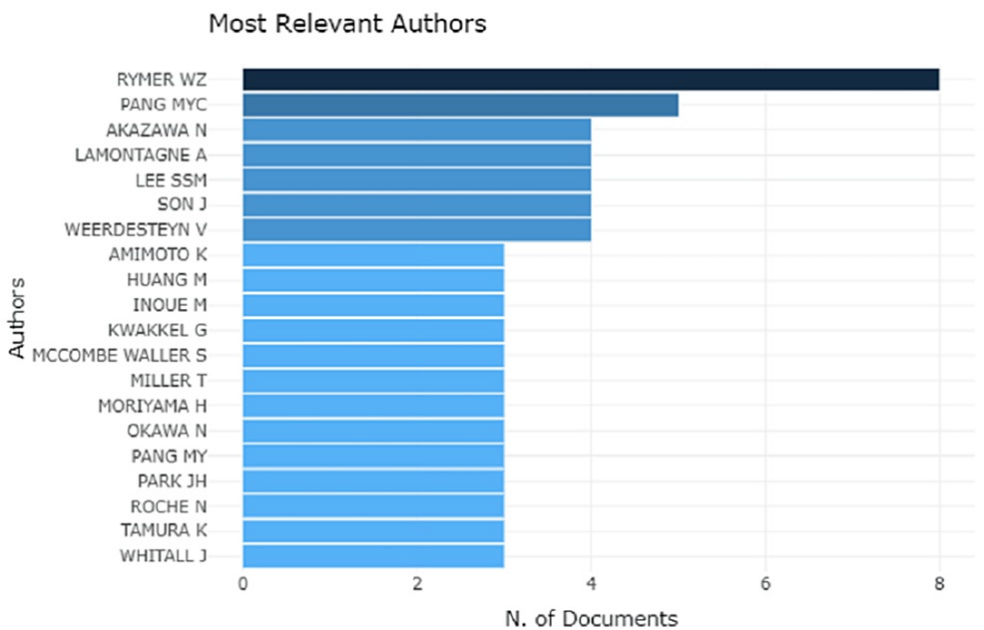
Most relevant authors with number of documents

Authors' knowledge maps and co-cited authors' knowledge maps can provide useful information on influential research teams and potential collaborators, allowing researchers to build collaborations. The 723 publications were written by a total of 611 authors. Figure 1 shows Rymer WZ ranked first with regard to publication, as a number of documents published were eight, followed by Pang MYC, Akazawa N, Lamontagne A, Lee Smm, Son J, and Weerdesteyn V had scored between two to six while 13 other authors had to score less than two.

Analysis of Institutions and Countries

**Figure 2:**
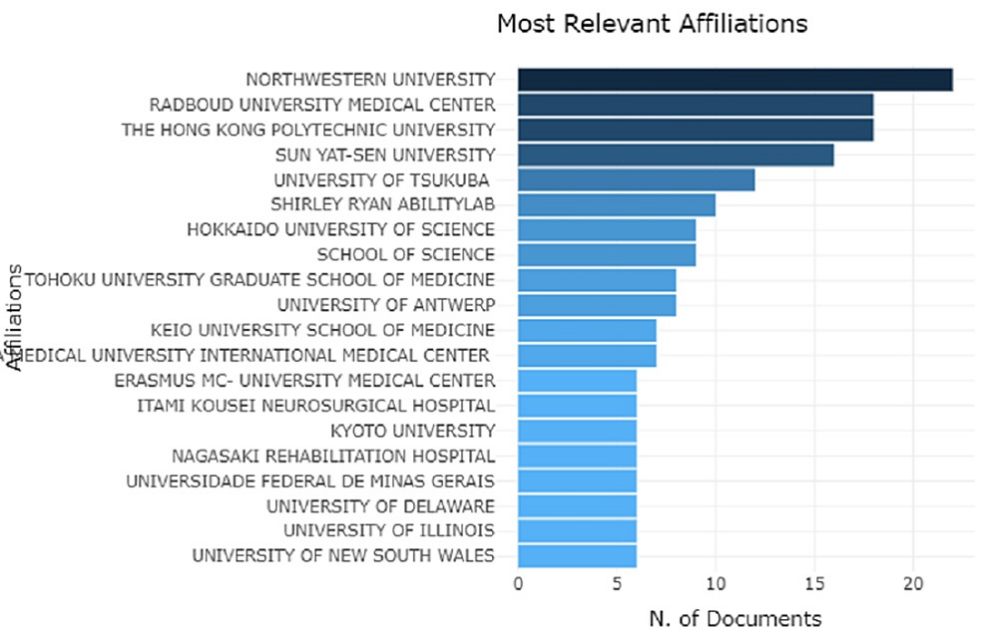
Graphical representation of universities with most relevant affiliations

The most relevant affiliations with a maximum number of articles are from Northwestern University, followed by Radboud University Medical Center and The Hong Kong Polytechnic University with a minimum number from the University of New South Wales, as shown in Figure 2.

**Figure 3:**
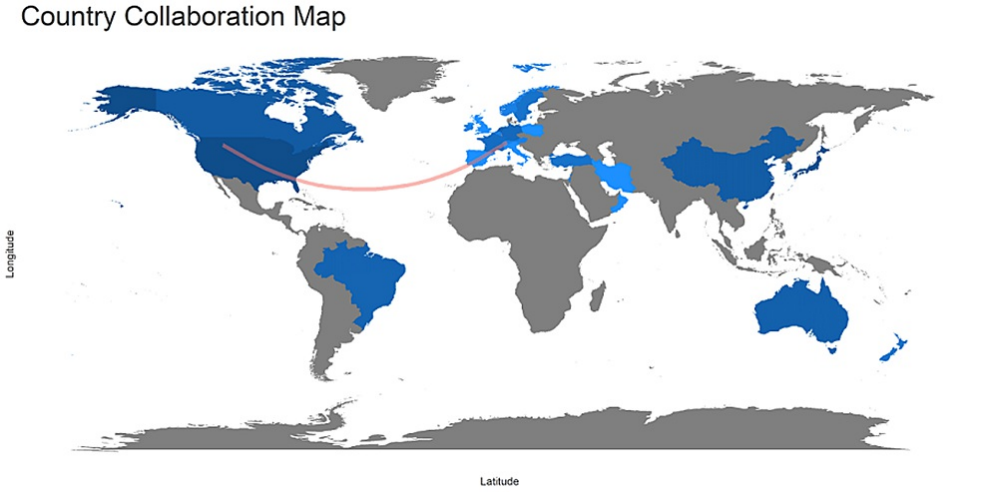
Pictorial map representing the scientific production of the country

The pictorial map represents the scientific production of the country, showing a high number of researchers from the United States, followed by Spain, France, and Germany, as shown in Figure 3.

Analysis of Journal and Its Growth Trend

**Figure 4:**
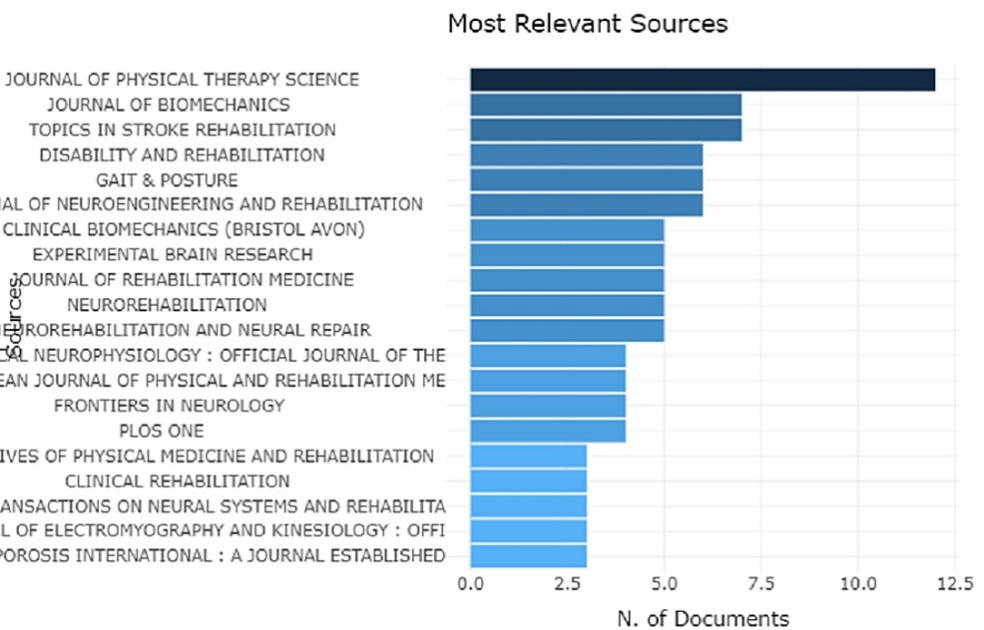
Graphical representation of most relevant sources with a number of documents published

The Journal of Physical therapy science has the most relevant articles which are more than 10. While Journal in Biomechanics, Topics in Stroke Rehabilitation, Disability and Rehabilitation, Gait and Posture, and Journal of Neuro-engineering and Rehabilitation have an article count between five to 7.5. Rest 13 journals have a count of less than five as shown in Figure 4.

**Figure 5:**
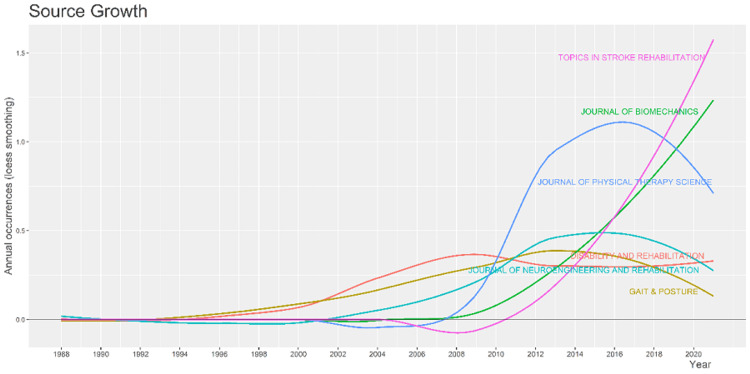
Growth rate of the journals

The growth trend of topics in stroke rehabilitation showed a tremendous increase from 2008 and is at the top to date while that of the gait and posture decreased from 2018. It is well represented in Figure 5.

Analysis of Research Database According to Bradford’s Law

**Figure 6:**
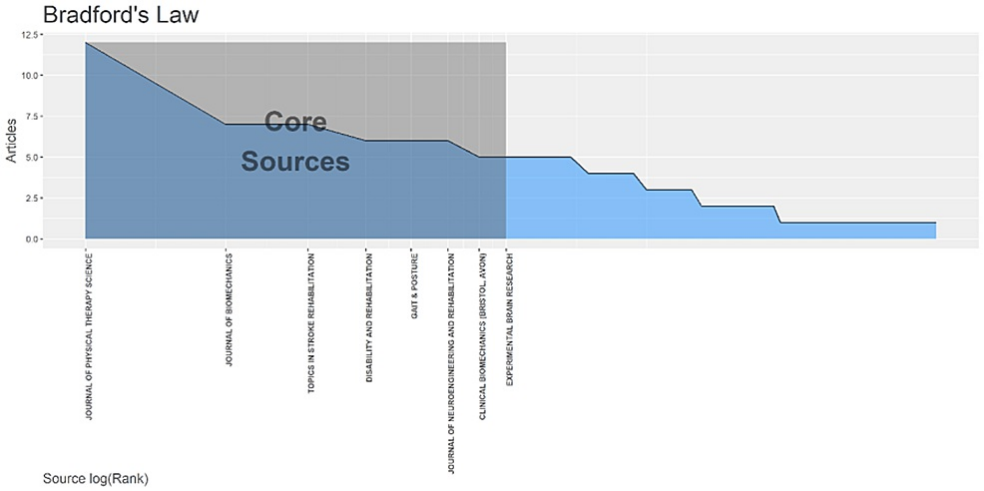
Bradford’s law representing core source of research

Bradford's law analysis provides us with the number of core articles and Bradford's multiplier that a researcher requires when a dozen or more articles are required for the study. It is provided in Figure 6.

Analysis of Citations and Authors' Publications

**Figure 7:**
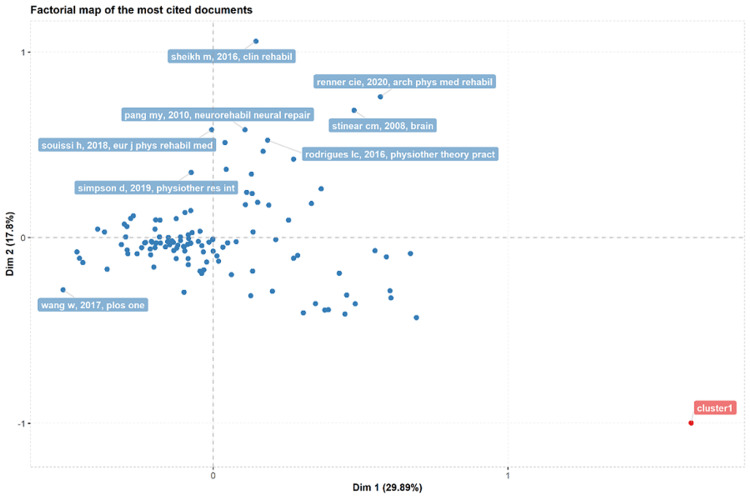
Factorial representation of most cited documents

One of the most important aspects of scientific research is the number of cited articles among the total number of published articles. The more the number of citations is the better the quality. Here, Sheikh m,2016, Clinical Rehabilitation has the most cited articles along with Renner Cie, 2020, Archives in Physical Medicine and Rehabilitation, as represented in Figure 7.

**Figure 8:**
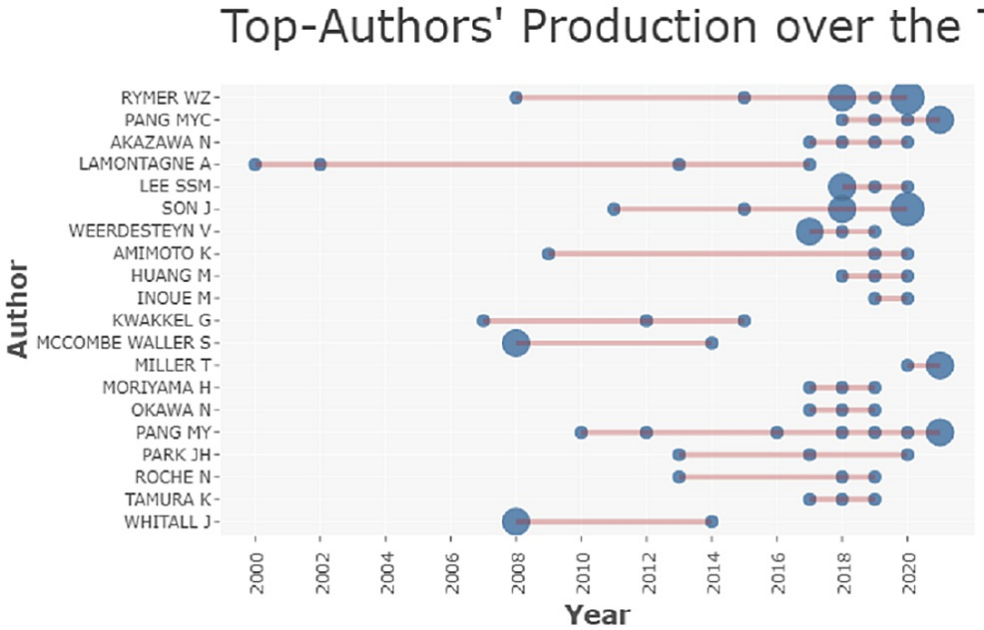
Top author production from 2000 to 2020.

The top author’s production of research over the period of 20 years is shown in Figure 8, with Rymer being at the top while Whitall being at the bottom.

Analysis of Study Concept and Trends

**Figure 9:**
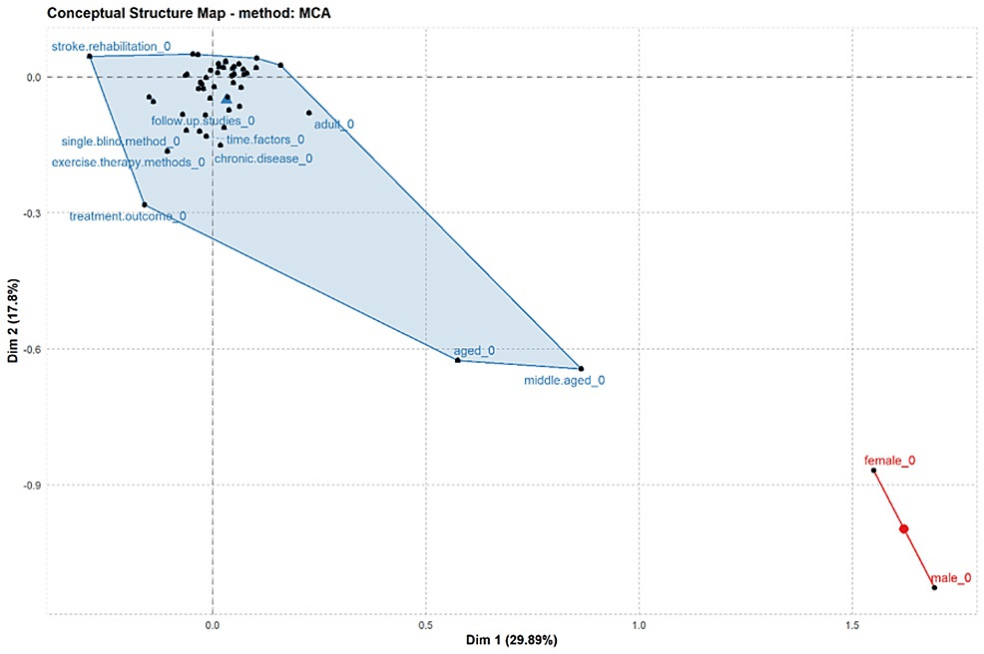
Conceptual structural map

The domains and diverse elements of studies undertaken on bilateral training in stroke patients, stroke rehabilitation, dynamic gait index, Fugl Mayer assessment, and follow-up studies are shown in the conceptual structure map in Figure 9.

Analysis of Words

**Figure 10:**
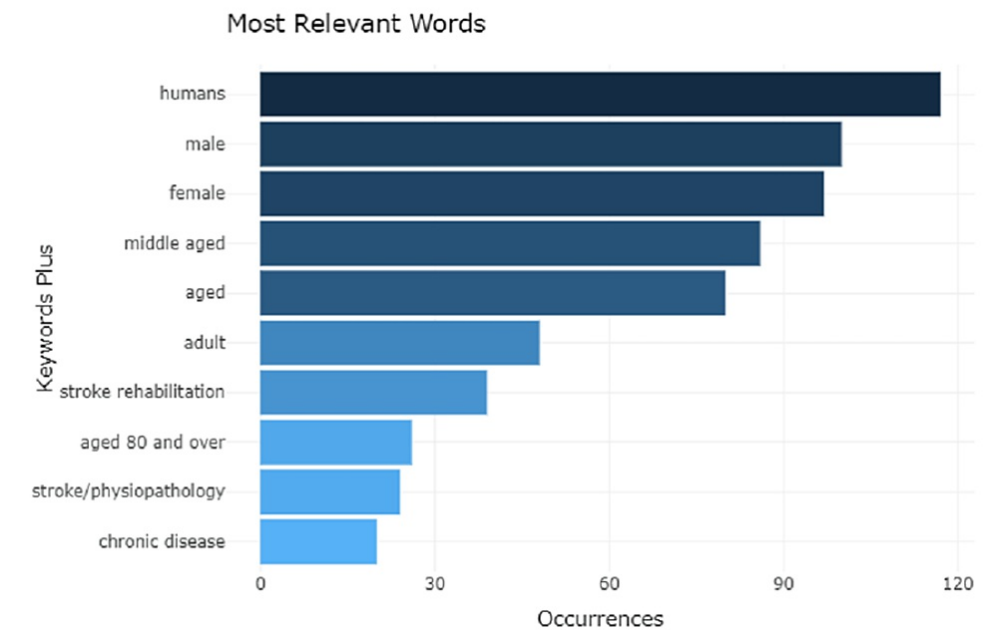
Most frequently used words with their frequency

The study reveals that the most frequently used word is human with a frequency of 118, followed by the male, female, and middle-aged with consecutive decreasing frequency. The last occurrence is of the word chronic disease with a frequency of 27, as shown in Figure 10.

**Figure 11:**
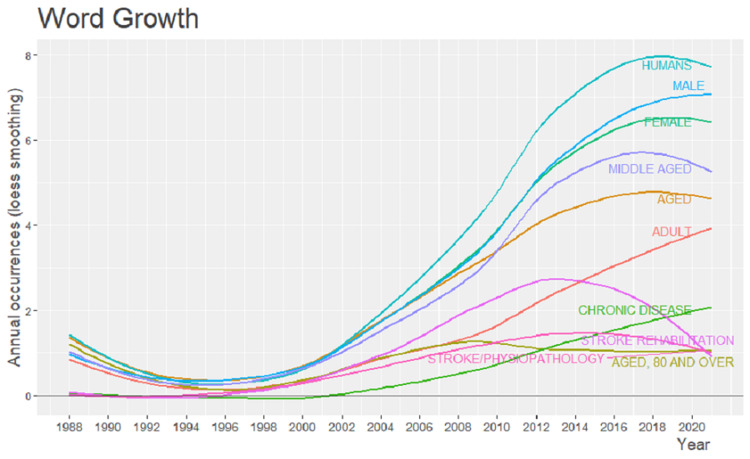
Growth trend of most frequently used words

The growth rate in word frequency has increased over time. From 1988 to 2020, the human word was at its peak i.e., eight, followed by male and female being seven and 6.5 respectively. Initially, there was no usage of stroke pathophysiology from the year 1998 to 1996, with a gradual rise till the year 2012, there was a fall in the last few years. Similarly, there is a fall in stroke rehabilitation in 2016. It is represented in Figure 11.

A large network exists between the most commonly used words globally throughout all research publications, as shown by a visual depiction derived from the study.

Discussion

Stroke-causing disability in the form of hemiparesis and hemiplegia can lead to long-term dependency. To reduce that disabling condition, early management of the cause and the secondary impairment is of utmost importance. This review of international research in the fields of stroke rehabilitation and hemiplegic balance and walking using a bibliometric method revealed a rise in the number of papers published in the last two decades. The retrieval method yielded 160 objective results. CiteSpace software was used to extract bibliometric indicators (keywords, countries, institutions, and other objects) from the data. Processing with the analysis tools resulted in relational figures, tables, and data interpretation. Even if the number of research publications published on this subject is lower than in other medical fields, there is a trend that indicates an increase in total research.

A total of 611 author lists were generated, with only one author for single-authored documents and 610 for multi-authored publications. Journal of Physical Therapy Science had the most relevant articles (almost 12) followed by Journal of Biomechanics, Topics in Stroke Rehabilitation, Disability and Rehabilitation, Gait and Posture, and Journal of Neuro Engineering and Rehabilitation all have between 5 and 7.5 articles each. It is important to note that this increase in the literature does not imply an improvement in the quality of the papers, as the data extraction procedure could only determine the number of papers published in this sector and not their quality. The fact that some articles have a high number of citations simply means that academics have paid more attention to them.

A maximum number of articles found have shown the improvement in hand function with bilateral training and its relationship with strengthening, which laid down the pillars for a similar study on the lower limb. Other articles showed the improvement that the patients had post-stroke rehabilitation was comparatively enormous as compared to those who do not undergo a particular rehabilitation regime [21,22]. Researchers have proposed a personalized rehabilitation program, which stresses prescribing an exercise regimen based on a patient's needs, preferences, and skills, in light of the growing belief that "rehabilitation in stroke is medicine."

Jeon et al. in their control trial in the stroke population, found bilateral training to be more effective than unilateral alone, as the uninvolved side training is equally important to improve balance and gait [23]. This study showed the positive effects of strengthening balance. This study will provide a further view of the patient’s quality of life post-discharge [24,25].

Strength and limitation

This is the first bibliometric analysis based on PubMed data to describe the development and trends of global scientific research into lower limb rehabilitation after stroke over the last two decades. The 160 papers came from 22 scholarly journals and supplemented the findings. This study looked at subject categories, references, authors, and key terms in addition to the number of publications, citations, journals, and cooperation across countries/institutions/authors.

There are several limitations to this study as well. To begin, the retrieval approach was limited to PubMed core databases, with non-English papers being excluded. As a result of these circumstances, there may be a publishing bias. Second, the study did not use CiteSpace software for geospatial visualization; yet this had no effect on our findings. The third constraint was that some key publications may not have received many citations, while others may have been mentioned frequently enough for their findings to be widely known.

## Conclusions

This review of studies on exercise therapies for stroke survivors published over the previous two decades could be valuable in establishing better hemiplegia rehabilitation programs. This study could help research teams collaborate to promote the use of strengthening in the therapeutic management of hemiplegia balance. There are also other high-quality randomized controlled trials in progress. Despite its limitations, this study gives historical insight into stroke rehabilitation and provides researchers with information on potential collaborations with other institutions and academics, popular subjects, and development trends.

## References

[1] (2021). World Health Organization (WHO) Definition of Stroke. https://www.publichealth.com.ng/world-health-organization-who-definition-of-stroke/.

[2] Physical Rehabilitation - Susan B O’Sullivan, Thomas J Schmitz (2022). Physical rehabilitation. https://books.google.co.in/books?hl=en&lr=&id=Vs6FDwAAQBAJ&oi=fnd&pg=PR3&dq=Physical+Rehabilitation,+6th+edition+sullivan&ots=DkGnWO_JbV&sig=-eQM-KYHLMcRYtc8wHTng721bq4&redir_esc=y#v=onepage&q=Physical%20Rehabilitation%2C%206th%20edition%20sullivan&f=false.

[3] Khurana D, Padma MV, Bhatia R (2018). Recommendations for the early management of acute ischemic stroke: a consensus statement for healthcare professionals from the Indian Stroke Association. J Stroke Med.

[4] Carin-Levy G, Greig C, Young A, Lewis S, Hannan J, Mead G (2006). Longitudinal changes in muscle strength and mass after acute stroke. Cerebrovasc Dis.

[5] Adams RW, Gandevia SC, Skuse NF (1990). The distribution of muscle weakness in upper motoneuron lesions affecting the lower limb. Brain.

[6] Kamalakannan S, Gudlavalleti AS, Gudlavalleti VS, Goenka S, Kuper H (2017). Incidence & prevalence of stroke in India: a systematic review. Indian J Med Res.

[7] Banerjee TK, Das SK (2016). Fifty years of stroke researches in India. Ann Indian Acad Neurol.

[8] Sjöström M, Fugl-Meyer AR, Nordin G, Wählby L (1980). Post-stroke hemiplegia; crural muscle strength and structure. Scandinavian J Rehab Med.

[9] Wist S, Clivaz J, Sattelmayer M (2016). Muscle strengthening for hemiparesis after stroke: a meta-analysis. Ann Phys Rehabil Med.

[10] Davidoff RA (1990). The pyramidal tract. Neurology.

[11] Teixeira-Salmela LF, Olney SJ, Nadeau S, Brouwer B (1999). Muscle strengthening and physical conditioning to reduce impairment and disability in chronic stroke survivors. Arch Phys Med Rehabil.

[12] Mudie MH, Matyas TA (2000). Can simultaneous bilateral movement involve the undamaged hemisphere in reconstruction of neural networks damaged by stroke?. Disabil Rehabil.

[13] Morris SL, Dodd KJ, Morris ME (2004). Outcomes of progressive resistance strength training following stroke: a systematic review. Clin Rehabil.

[14] Ada L, Dorsch S, Canning CG (2006). Strengthening interventions increase strength and improve activity after stroke: a systematic review. Aust J Physiother.

[15] Yang YR, Wang RY, Lin KH, Chu MY, Chan RC (2006). Task-oriented progressive resistance strength training improves muscle strength and functional performance in individuals with stroke. Clin Rehabil.

[16] Chen PM, Kwong PW, Lai CK, Ng SS (2019). Comparison of bilateral and unilateral upper limb training in people with stroke: a systematic review and meta-analysis. PLoS One.

[17] Wu J, Cheng H, Zhang J, Bai Z, Cai S (2021). The modulatory effects of bilateral arm training (BAT) on the brain in stroke patients: a systematic review. Neurol Sci.

[18] Kanase S (2020). Effect of Motor relearning programme and conventional training on functional mobility in post stroke patients. Chin. J. Contemp. Neurol. Neurosurg.

[19] Johannsen L, Wing AM, Pelton T (2010). Seated bilateral leg exercise effects on hemiparetic lower extremity function in chronic stroke. Neurorehabil Neural Repair.

[20] Todd JS, Shurley JP, Todd TC (2012). Thomas L. DeLorme and the science of progressive resistance exercise. J Strength Cond Res.

[21] McCombe Waller S, Whitall J, Jenkins T, Magder LS, Hanley DF, Goldberg A, Luft AR (2014). Sequencing bilateral and unilateral task-oriented training versus task oriented training alone to improve arm function in individuals with chronic stroke. BMC Neurol.

[22] Whitall J, McCombe Waller S, Silver KH, Macko RF (2000). Repetitive bilateral arm training with rhythmic auditory cueing improves motor function in chronic hemiparetic stroke. Stroke.

[23] Jeon HJ, Hwang BY (2018). Effect of bilateral lower limb strengthening exercise on balance and walking in hemiparetic patients after stroke: a randomized controlled trial. J Phys Ther Sci.

[24] Liu M, Chen J, Fan W (2016). Effects of modified sit-to-stand training on balance control in hemiplegic stroke patients: a randomized controlled trial. Clin Rehabil.

[25] Samal SN, Samal SS, Ingale N, Chaudhary S, Gawande V (2021). Efficacy of core strengthening exercises on Swissball versus conventional exercises for improving trunk balance in hemiplegic patients following stroke. Int J Res Pharm Sci.

